# Embolic stroke complicating *Staphylococcus aureus *endocarditis circumstantially linked to rectal trauma from foreign body: a first case report

**DOI:** 10.1186/1471-2334-5-42

**Published:** 2005-05-27

**Authors:** Braj B Pandey, Tuan C Dang, John F Healy

**Affiliations:** 1 University of California, San Diego, USA

## Abstract

**Background:**

Diagnostic and therapeutic instrumentation of the lower gastrointestinal tract has been reported to result in bacteremia and endocarditis. No such case has been reported in persons with a history of rectal foreign body insertion despite its potential for greater trauma.

**Case presentation:**

A 58-year-old male was admitted with confusion and inability to speak. His past history was notable for hospitalization to extract a retained plastic soda bottle from the rectosigmoid two years prior. On examination, he was febrile, tachycardic and hypotensive. There was an apical pansystolic murmur on cardiac examination. He had a mixed receptive and expressive aphasia, and a right hemiparesis. On rectal examination he had perianal erythema and diminished sphincter tone. Magnetic resonance imaging of the brain showed infarction of the occipital and frontal lobes. Transesophageal Echocardiography of the heart revealed vegetations on the mitral valve. All of his blood culture bottles grew methicillin sensitive *Staphylococcus aureus*. He was successfully treated for bacterial endocarditis with intravenous nafcillin and gentamicin. The rectum is frequently colonized by *Staphylococcus aureus *and trauma to its mucosa can lead to bacteremia and endocarditis with this organism.

In the absence of corroborative evidence such as presented here, it is difficult to make a correlation between staphylococcal endocarditis and anorectal foreign body insertion due to patients being less than forthcoming

**Conclusion:**

There is a potential risk of staphylococcal bacteremia and endocarditis with rectal foreign body insertion. Further studies are needed to explore this finding. Detailed sexual history and patient counseling should be made a part of routine primary care.

## Background

There is a large body of surgical literature reporting anal eroticism resulting in rectal trauma and retained foreign bodies [1,2], but there is no report of bacteremia or endocarditis occurring in these patients. *Staphylococcus aureus *is an aggressive pathogen and bacteremia with this organism can infect healthy heart valves. The rectal mucosa is a major site of colonization by this organism. We describe a patient with a past history of surgical extraction of a retained plastic soda bottle from the rectosigmoid, who later developed staphylococcal endocarditis resulting in septic embolism and stroke. No such case has been reported in the literature.

## Case presentation

**Figure 1:**
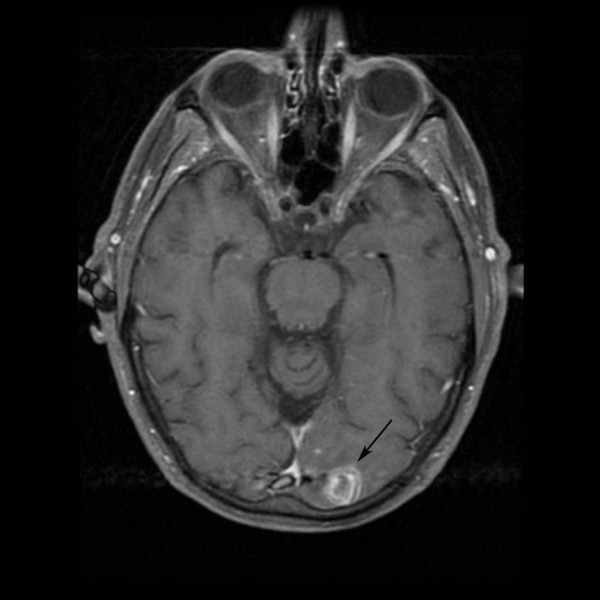
Contrast enhancing infarction in occipital lobe (arrow)

**Figure 2:**
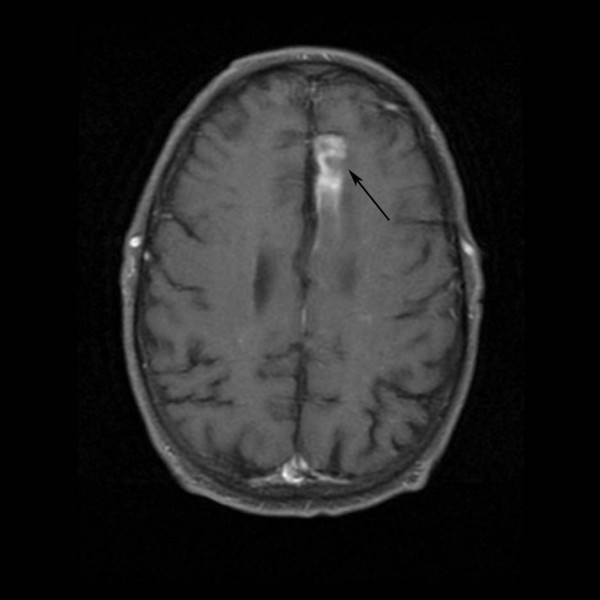
Contrast enhancing infarction in the frontal lobe (arrow).

A 58-year-old male was brought to the emergency room with confusion and an inability to speak for 1 day. He had a past history of hypertension and hypomania. He was single and he lived alone. On physical examination, his blood pressure was 80/63 mm of Hg, heart rate 126/minute, and temperature 102°F. He was awake but unable to speak due to a mixed receptive and expressive aphasia. He had right homonymous hemianopsia and hemiplegia. Cardiac examination was positive for a pansystolic murmur in the apical area. The abdominal examination was unremarkable. On rectal examination there was perianal erythema and diminished sphincter tone. Complete blood count showed WBC 15,400/mm^3 ^with a left shift, hematocrit 49%, and normal platelets. Serum chemistries showed glucose 84 mg/dl, albumin 2.9 mg/dl, and calcium 8.5 mg/dl. Magnetic Resonance Imaging of the brain, including diffusion weighted imaging, revealed acute hemorrhagic infarction of the left occipital lobe and acute embolic infarctions of the left frontal and right occipital lobes (Fig 1 and 2). Transesophageal Echocardiogram demonstrated mitral regurgitation and large vegetations on the posterior leaf of the mitral valve. Splenic and renal infarcts were visible on Computerized Tomography of the abdomen. All of the blood culture bottles and the urine culture grew methicillin sensitive *Staphylococcus aureus*. Treatment of the bacterial endocarditis was started with intravenous nafcillin and gentamicin. The patient had a significant recovery of speech and motor function within a few days. When asked about recent dental work, he gave a history of a tooth extraction 2–3 days before the hospitalization but was unable to provide information about his dentist. A dentist from the hospital examined the patient and found no clinical evidence of the extraction. The patient underwent a complete neuropsychiatric evaluation. He displayed confabulation and perseverance (marriage, retirement, hospitalization were reported using the same date which was his birthday). He also had significant executive dysfunction including concrete thinking and poor insight regarding his health and cognitive problems. On hospital day 12 the patient had mitral valve replacement surgery using a bioprosthetic valve. He completed a six-week course of intravenous antibiotic treatment. He also underwent extensive rehabilitation therapy and was sent home after 8 weeks of hospitalization. On subsequent follow-up visits the patient showed complete recovery from the stroke and was back to his baseline. In response to questions about his sexual history the patient indicated having heterosexual relations with multiple partners. However, his answers were inconsistent and seemed unreliable.

**Figure 3:**
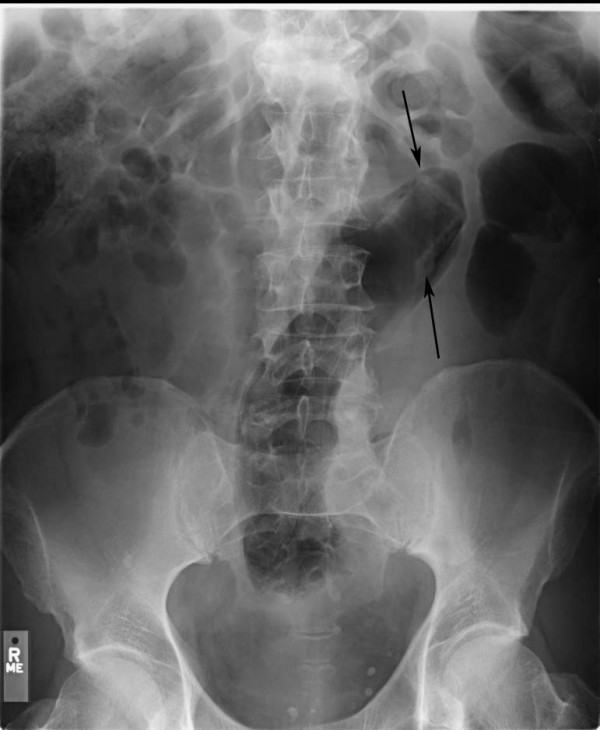
X-ray of abdomen 2 years prior showing outline of plastic soda bottle in sigmoid colon (arrows).

A closer examination of his medical records revealed that two years prior to this hospitalization, the patient was admitted with a plastic soda bottle retained in the rectosigmoid for 3 days. The bottle had been filled with warm water and inserted into his anal canal for sexual stimulation. It slipped all the way into the rectum and could not be retrieved. His attempts to extract it at home were unsuccessful. In the emergency room his physical examination was normal except for a palpable mass in the suprapubic area, decreased anal sphincter tone and a dilated rectal vault. On X-ray of the abdomen, the outline of a plastic bottle was visible in the rectosigmoid (Fig 3). The patient was taken to the operating room and under spinal anesthesia the bottle was extracted. He had multiple lacerations of the rectal mucosa but there was no perforation. He went home the next day. On follow-up visits to primary care he was noted to be overall healthy except having mild hypertension. His behavior was indicative of hypomania, but he did not get a formal psychiatric evaluation.

Foreign bodies in the rectum and methods of their extraction have been amply chronicled in the surgical literature [1]. Sexual stimulation is the reason in a majority of these cases [2]. Local trauma, perforation, and resultant peritonitis are well known complications [3]. An unlimited PUBMED search for articles on bacteremia or endocarditis related to rectal foreign body insertion was unfruitful (We tried MeSH terms anal/rectal/colorectal/foreign bodies/anorectal /sexual deviation for the purpose). Bacteremia and septicemia from barium enema [4], and therapeutic anal dilatation [5] have been published. Procedures like fiberoptic sigmoidoscopy are known to cause endocarditis [6], but septic stroke resulting from endocarditis related to lower gastrointestinal instrumentation has also not been reported.

The patient's history of anorectal insertion of a plastic soda bottle for sexual gratification is consistent with published reports of use of large objects for this purpose [7]. The resultant rectal trauma can easily lead to bacteremia. Rectal carriage of *Staphylococcus aureus *is well documented and is a potential source of infection [8]. This organism tends to be more abundant on the rectal mucosa than within the feces [9]. In a study of gastrointestinal colonization, *Staphylococcus aureus *grew from the culture of rectal swabs in 60% cases versus 53% positive culture of nasal swabs taken from the same subjects [10]. This organism is known to cause endocarditis of normal heart valves [11,12]. Neurologic complications of infective endocarditis, particularly embolic events, tend to be higher in cases of endocarditis caused by *Staphylococcus aureus *[13].

It is known that few patients with rectal foreign bodies will freely admit to transanal introduction [14]. This explains to some extent the paucity of literature linking this practice with bacteremia or endocarditis. We believe our patient was habituated to rectal insertion of foreign bodies and that is evident from his previous history along with the clinical findings of perianal erythema and diminished sphincter tone [14]. In the absence of a reliable history from the patient, the link between endocarditis and rectal trauma in this case is based on circumstantial evidence. A further study of patients with well-documented evidence of rectal foreign body insertion could be the next step to explore this important observation.

## Conclusion

The rectum is a frequent site of *Staphylococcus aureus *carriage. Trauma from foreign objects in the rectum carries a risk of staphylococcal bacteremia that is known to result in endocarditis of both normal and abnormal heart valves. Further studies are needed to explore this finding. It is important to get a detailed sexual history from patients visiting primary care clinics. Patient education and warning may help prevent catastrophic complications of this risky practice.

## Competing interests

The author(s) declare that they have no competing interests.

## Authors' contributions

BBP carried out the clinical study of the patient, conceived the study, researched the literature, and wrote the article. TCD carried out the clinical study of the patient, researched the literature, and edited the article. JFH provided radiological diagnosis, figure legends and computerized figures.

## Pre-publication history

The pre-publication history for this paper can be accessed here:


